# Relationship of serum estradiol and progesterone with symptoms and sex difference in schizophrenia: A cross-sectional study in Iran

**DOI:** 10.3389/fpsyt.2023.1075780

**Published:** 2023-03-08

**Authors:** Najmeh Shahini, Zanireh Salimi, Dorsa Kiani, Ahmad Raftari, Maliheh Ziaee

**Affiliations:** ^1^Golestan Research Center of Psychiatry (GRCP), Golestan University of Medical Sciences, Gorgan, Iran; ^2^Clinical Research Development Unit (CRDU), 5 Azar Hospital, Golestan University of Medical Sciences, Gorgan, Iran; ^3^Psychiatry and Behavioral Sciences Research Center, Mashhad University of Medical Sciences, Mashhad, Iran; ^4^Department of Community Medicine, School of Medicine, Social Determinants of Health Research Center, Gonabad University of Medical Sciences, Gonabad, Iran

**Keywords:** estradiol, progesterone, schizophrenia, symptom, syndrome

## Abstract

**Background:**

Schizophrenia is a devastating disease characterized by frequent relapses, cognitive decline, and emotional and functional disability, with unknown causes. The phenomenology and clinical course of schizophrenic disorders are different between the two genders, which is thought to be related mainly to the effects of steroid sex hormones on the nervous system. Regarding inconsistencies in the studies, we aimed to compare the levels of estradiol and progesterone between schizophrenia patients and healthy individuals.

**Methods:**

This cross-sectional study was conducted on 66 patients referred to the specialized clinical psychiatric ward of a teaching hospital in the north of Iran, for 5 months in 2021. Thirty-three schizophrenia patients confirmed by a psychiatrist based on DSM5 criteria were included in the case group, and 33 individuals without a psychiatric disease were included in the control group. We completed a demographic information checklist for each patient, along with the Simpson-Angus extrapyramidal side effect scale (SAS) for drug side effects and the positive and negative syndrome scale (PANSS) for the severity of the disease symptoms. Then, a 3-ml blood sample was taken from each participant to determine the serum levels of estradiol and progesterone. The data were analyzed by SPSS16 software.

**Results:**

Thirty-four (51.5%) and 32 (48.5%) participants in this study were male and female, respectively. The mean serum level of estradiol was 22.33 ± 13.65 pm/dl in schizophrenia patients and 29.36 ± 21.32 pm/dl in the control group, showing no significant difference between the two groups (*P* = 0.4). However, the mean serum level of progesterone was significantly lower in schizophrenia patients (0.37 ± 1.39 pm/dl) than in control subjects (3.15 ± 5.73 pm/dl) (*P* < 0.001). The PANSS and SAS scores were not significantly correlated with the level of sex hormones (*P* > 0.05). Serum estradiol and progesterone levels based on sex significantly differed between the two groups (except for female estradiol).

**Conclusion:**

Considering the hormonal differences between schizophrenia patients and control subjects, determining hormonal levels in these patients and using complementary hormonal therapies with estradiol or similar compounds can be beneficial as the starting point of schizophrenia treatment, where therapeutic responses can draw the future developmental framework.

## Introduction

Schizophrenia is a devastating disease characterized by frequent relapses, cognitive decline, and emotional and functional disability. This disorder encompasses positive (hallucinations, delusions) and negative (dark emotions, apathy) symptoms and is recognized as a cognitive disorder highly resistant to pharmaceutical treatment (1–3). Schizophrenia is a severe psychiatric disorder of unknown etiology. Many fundamental studies have been conducted to divulge the biological processes of the disease and understand its possible etiologies. The phenomenology and clinical course of schizophrenic disorders are also different between the two genders, (4) which is thought to be primarily related to the effects of steroid sex hormones on the nervous system (5, 6). Women with schizophrenia have less functional dysfunction, milder symptoms, and relatively late-onset disease. Studies have shown that estradiol can have a protective role against this disease. Also, progesterone belongs to another group of neuroactive steroids that binds to and activates progesterone receptors (7).

Endogenous progesterone (P4) is humans’ most potent and abundant hormonal subgroup. This hormone is synthesized both centrally and peripherally from pregnenolone, a cholesterol-derived steroid precursor. Progesterone (a precursor of estrogens), testosterone (the primary gonadal hormone in men), and various types of glucocorticoids and mineralocorticoids play an essential role in the health and growth of men and women. It has been reported that serum progesterone levels range between 1 and 3 nM in men, with slight fluctuations with age. On the other hand, premenopausal women experience a variable serum level of progesterone according to the menstrual cycle. Accordingly, the lowest concentration of progesterone has been reported in the early follicular phase (0.3–2 nM) and the highest in the middle of the luteal phase. The serum level of progesterone drops considerably after menopause (3, 8).

Estradiol is the most potent endogenous estrogen in humans and the main focus of this study. Estrogens are synthesized both centrally and peripherally. Estradiol is a hormone that exists in both men and women; however, its serum levels are lower in adult men than in women, showing the least age-related fluctuations. The lowest and highest estradiol levels are seen in the early follicular stage (an average level of 150 pM) and on the day of ovulation (an average level of 670 pM), respectively. In the middle of the luteal phase, estradiol shows an average level of 500 pM (9). In general, women show higher levels and fluctuations of estradiol and progesterone than men. While progesterone exerts its effects primarily through the activation of its receptors, it also promotes significant indirect effects after being converted into some neuroactive molecules, including testosterone and estrogens (10, 11). It seems that estrogen acts as an agonist in the serotonergic system and affects serotonin transmission between neurons by suppressing the activity of monoamine oxidase (12), the catabolizing enzyme of serotonin and dopamine. In turn, increased serotonin levels in the synapse lead to possible mood change (13). Estrogen also plays a role in neurotransmitter systems, amplifies the activity of noradrenaline, acts as a cholinergic agonist, and possibly decreases sensitivity to the D2 dopamine receptor (14).

While studies consider a role for the fluctuations of sex gonadal hormones in the phenomenology of schizophrenia in women and men, there is little research on the profiles of gonad hormones and neurosteroids in this disorder (15). Sun et al. suggested progesterone as a neglected hormone in schizophrenia, declaring a role for this hormone in patients’ symptoms, predominantly negative symptoms, and its relationship with estradiol levels (16), opening spaces for further studies. Low estrogen levels increase the rate of disorders such as irregular menstruation in women with schizophrenia, which has been attributed to antipsychotic-induced hyperprolactinemia. However, there is evidence negating the occurrence of “hypoestrogenism” in schizophrenic women merely treated with antipsychotics causing hyperprolactinemia. Although the exact mechanism underlying the low levels of estrogen in schizophrenic women is yet to be disclosed, “hypoestrogenism” is of clinical importance because estrogen seems to exert an antipsychotic effect and positively affect the disease process in schizophrenic women. Compared to healthy individuals, the serum levels of estradiol decline in women with schizophrenia before menopausal and menstruation. In addition, the decreased plasma progesterone levels in the luteal phase indicate ovulatory cycles and insufficient follicle maturation. Higher average estrogen levels have been associated with better neuropsychological performance in various cognitive domains, including executive performance, verbal memory, spatial memory, focus/speed, and global cognitive function. In healthy people, estrogen seems to have a more specific relationship with verbal memory and fluency. There are inconsistencies between the studies assessing the effects of sex hormones on schizophrenia (17–19).

Besides, estrogen, estradiol, and progesterone levels differ between men and women, and a few studies have been conducted on this issue in Iran. Therefore, we aimed to compare estradiol and progesterone levels between patients with schizophrenia and healthy individuals.

## Materials and methods

This cross-sectional study was performed on patients referred to specialized psychiatric clinics and ward of the Martyrs of 5th Azar Teaching and Medical Center in Gorgan (north of Iran) for 5 months in 2021.

### Participants

The participants were included in this study by convenience method sampling. A total of 72 people entered the study. Three persons from the case group were excluded. After some participants were withdrawn from the study, in total, this study was performed on 66 participants. Thirty-three people in the case group were selected based on DSM5 criteria and with the approval of a psychiatrist, and 33 people in the control group were selected from patients referred to the psychiatric wards of a teaching hospital (Martyrs of 5th Azar) in Gorgan who did not have a psychiatric illness. This hospital is a specialty hospital with a psychiatric ward.

The inclusion criteria were the age of 18 to 50, schizophrenia diagnosis based on DSM5 criteria by a specialist, and admission during the luteal phase (for women). The inclusion criteria in the control group encompassed people visiting the laboratory without a psychiatric illness. The exclusion criteria included a history of taking Oral Contraceptive Pills (OCP) or hormonal contraceptives in the last 3 months, a history of other neurological disorders requiring treatment, the presence of polycystic ovaries or other diseases with hormonal changes such as hyperprolactinemia, Edison, or tumor, postmenopausal and pregnant women, lactating mothers 3 months after delivery, and those not satisfied with the study.

### Sample size

A sample size of 70 people was calculated based on the Yazici et al. study (20) with a test power of 80% and α = 0.05. We considered 72 people to enter the study (possible attrition of participants was considered):


$$n=\frac{\left(s_{1}^{2}+s_{2}^{2}\right)*{\left(z_{1-\frac{\alpha }{2}}+z_{1-\beta }\right)}^{2}}{d^{2}}.$$

### Psychiatric evaluation

A checklist was used for each patient to record demographic characteristics and serum levels of estradiol and progesterone. This checklist included multi-digit codes, age, sex, education, height, weight, medical history, history of psychiatric disorders and frequency of episodes, history of extrapyramidal complications, history of Electro convulsive Therapy (ECT), history of drug use, and history of supplement use. The positive and negative syndrome scale (PANSS) was used to assess the severity of positive and negative symptoms in schizophrenic patients, with three subscales: positive symptoms with seven questions, negative symptoms with seven questions, and general symptoms with 16 questions. This questionnaire’s validity and reliability have been confirmed by Ghamari Givi (21). The Simpson-Angus extrapyramidal side effect scale (SAS) was used to assess the severity of extrapyramidal side effects of schizophrenia in patients, which was completed by the examiner. It included ten questions and symptoms scored on a five-point scale from 0 to 4. The sum of the scores obtained from the questionnaire was used. The validity and reliability of this questionnaire have been confirmed by Simpson and Angus (22).

### Blood collection

After obtaining informed consent from each person (informed consent was obtained from the patient or her/his legal guardian), 3 ml of blood was taken and transferred to the laboratory in ice boxes as soon as possible. The hormone levels were measured with micrometer accuracy by immunoassay (4) or Enzyme-linked Immunosorbent Assay (ELISA) method. The US-licensed Chinese Estradiol AccuBind Microplate ELISA Test system kit was used to measure serum estradiol levels, and the Progesterone AccuBind ELISA test system kit to measure serum progesterone levels. Based on the criteria set by kit instructions, the male participants were divided into three groups based on the estradiol level: less than 4, between 4 and 94, and more than 94, representing deficient, normal, and high levels, respectively. In women, levels less than 44 showed estradiol deficiency, and in the luteal phase, 44–196 is the normal range in this phase, and above 196 indicated increased estradiol. Also, according to the criteria set by the kit, men were divided based on the progesterone level into normal (0.1–1.2) and above normal groups (> 1.2), while women were grouped into normal (2–25) and high (> 25).

### Statistical analysis

Descriptive and inferential statistics were applied. The independent *t*-test (for two groups) or ANOVA test (for more than two groups) were performed to compare estradiol and progesterone data with normal distribution; otherwise, Mann–Whitney or Kruskal–Wallis test was used. The effect size (Cohen’s D) was also performed in two groups, interpreted as trivial (0–0.19), small (0.5–0.79), moderate (0.5–0.79), and large (> 0.8) (23). The Pearson correlation test assessed the relationship between age, SAS, and PANSS scores and estradiol and progesterone categories. The statistical analyses were done with SPSS16. The study followed the STROBE checklist, designed to ascertain the high quality of reports of observational studies.

## Results

We selected 35 people for the case group based on DSM5 criteria confirmed by a psychiatrist and 37 people for the control group. Three persons from the case group were excluded due to dissatisfaction with the study, sampling error, and coronary heart disease. In total, this study was performed on 66 participants.

The demographic characteristics, including sex, race, marital status, history of ECT, and opium use, were statistically different between the two groups. Table 1 presents the demographics as mean ± SD and numbers (percentages).

**TABLE 1:** Baseline characteristics of study participants, including demographic data.

Characteristics	Categorization	Case *N* (%)	Control *N* (%)	*P*-value
Age M (SD)		39.78 (9.54)	40 (14.57)	0.944**
Sex *N* (%)	Male	25 (75.8)	9 (27.3)	< 0.001*
	Female	8 (24.2)	24 (72.7)	
Education *N* (%)	Below diploma	17 (51.5)	11 (33.3)	0.117*
	Diploma	13 (39.4)	13 (39.4)	
	Above diploma	3 (9.1)	9 (27.3)	
Race *N* (%)	Turkman	4 (12.1)	0	0.012^¥^
	Fars	20 (60.6)	31 (93.9)	
	Sistani	6 (18.2)	1 (3)	
	Other	3 (9.1)	1 (3.1)	
Marriage *N* (%)	Single	19 (57.6)	27 (81.8)	0.001*
	Married	14 (42.4)	6 (18.2)	
Blood group *N* (%)	A^+^	6 (18.2)	11 (33.3)	0.729^¥^
	A^–^	4 (12.1)	1 (3)	
	B^+^	5 (15.2)	6 (18.2)	
	B^–^	3 (9.1)	2 (6.1)	
	O^+^	11 (33.3)	9 (27.3)	
	O^–^	1 (3)	2 (6.1)	
	AB^+^	2 (6.1)	1 (3)	
	AB^–^	1 (3)	1 (3)	
Past medical history *N* (%)	Yes	3 (9.1)	1 (3)	0.307^¥^
History of ECT *N* (%)	Yes	19 (57.6)	0	< 0.001^¥^
Cigarette use *N* (%)	Yes	4 (12.1)	0	0.057^¥^
Opium use *N* (%)	Yes	11 (33.3)	0	< 0.001^¥^

*Chi- squared test.

**Independent sample *t*-test.

^¥^Fisher’s exact test.

*N* (%), number (percent);

M (SD), mean (standard deviation).

### Serum estradiol and progesterone levels in the schizophrenic group

Although serum estradiol levels were lower in the schizophrenic group than in the control group, it was insignificant due to a small effect size. However, the progesterone level was statistically different between the two groups with a medium effect size (Table 2).

**TABLE 2:** Means of estradiol, progesterone and questionnaire scales in participants.

	Case M (SD)	Control M (SD)	Minimum case	Maximum case	Minimum control	Maximum control	*P*-value	Cohen’s D
Estradiol	22.33 (13.65)	29.36 (21.32)	1.27	49.1	4.7	85.45	0.48^£^	0.39
Progesterone	0.37 (1.39)	3.15 (5.73)	0.1	8.17	0.1	21.18	< 0.001^€^	0.66
SAS total	2.93 (2.12)	–					–	
PPANSS	30.51 (4.24)	–					–	
NPANSS	22.03 (5.2)	–					–	
GPANSS	49.87 (7.07)	–					–	
PANSS total	102.42 (12.73)	–					–	

^£^Independent samples *t*-test.

^€^ Mann–Whitney U test.

M (SD), mean (standard deviation).

A power analysis was conducted using G*Power version 3.1.9.7 for relation between estradiol level in two groups (not statistically significant), based on data from study. With a significance criterion of α = 0.05 and effect size = 0.39 and sample size = 33 in each group, the obtained power was been 34%.

Table 3 presents the categories of progesterone and estradiol serum levels in the two groups. Estradiol and progesterone serum levels based on sex were statistically different between the two groups (except for female estradiol).

**TABLE 3:** Categorical progesterone and estradiol serum levels in two groups.

	Categorization	Case *N* (%)	Control *N* (%)	*P*-value*
Male estradiol	Deficiency	6 (18.2)	0	0.012*
	Normal	27 (81.8)	33 (100)	
	Above normal	0	0	
Female estradiol	Deficiency	31 (93.9)	26 (78.8)	0.074*
	Normal	2 (6.1)	7 (21.2)	
	Above normal	0	0	
Male progesterone	Deficiency	17 (85)	6 (26.1)	< 0.001*
	Normal	2 (10)	6 (26.1)	
	Above normal	1 (5)	11 (47.8)	
Female progesterone	Deficiency	32 (97)	24 (72.7)	0.006*
	Normal	1 (3)	9 (27.3)	
	Above normal	0	0	

*N* (%), number (percent).

*Fisher’s exact test.

The questionnaire scores were interrelated, but no significant correlation (Pearson or Spearman) was found between the estradiol and progesterone levels and any of the questionnaire scores (Table 4).

**TABLE 4:** Correlation between SAS and PANSS scores and progesterone and estradiol.

	Sum of SAS questionnaire		Sum of PPANSS questionnaire		Sum of NPANSS questionnaire		Sum of GPANSS questionnaire		Sum of total PANSS questionnaire		Estradiol		Progesterone	
	***r* ^¥^**	*P* *	***r* ^¥^**	*P* *	***r* ^¥^**	*P* *	***r* ^¥^**	*P* *	***r* ^¥^**	*P* *	***r* ^¥^**	*P* *	***r* ^¥^**	*P* *
Sum of SAS questionnaire	1		0.167	0.354	0.196	0.275	0.193	0.281	0.243	0.173	0.119	0.508	−0.169	0.347
Sum of PPANSS questionnaire	0.167	0.354	1		0.125	0.488	0.191	0.288	0.49	0.004	0.07	0.67	−0.24	0.178
Sum of NPANSS questionnaire	0.196	0.275	0.125	0.488	1		0.678	< 0.001	0.897	< 0.001	−0.307	0.882	−0.208	0.244
Sum of GPANSS questionnaire	0.193	0.281	0.191	0.288	0.678	< 0.001	1		0.897	< 0.001	−0.027	0.882	−0.178	0.321
Sum of total PANSS questionnaire	0.243	0.173	0.49	0.004	0.827	< 0.001	0.897	< 0.001	1		−0.115	0.523	−0.264	0.137
Progesterone	−0.169	0.347	−0.24	0.178	−0.208	0.244	−0.178	0.321	−0.264	0.137	0.24	0.178	1	

^¥^Correlation coefficient.

*Pearson or Spearman tests.

We did not find a statistically significant association between categorized estradiol and progesterone levels in men and women and questionnaire scores. Because of the non-normal distribution of estradiol and progesterone data, we performed the Kruskal–Wallis test (Table 5).

**TABLE 5:** Association of progesterone and estradiol serum levels with scores of questionnaires.

	*P*-value* SASS total	*P*-value* PPANSS	*P*-value* NPANSS	*P*-value* GPANSS	*P*-value* PANSS total
Estradiol male	0.492	0.182	0.59	0.198	0.242
Estradiol female	0.378	0.705	0.733	0.173	0.273
Progesterone male	0.501	0.2	0.339	0.409	0.289
Progesterone female	0.286	0.14	0.205	0.206	0.155

*Kruskal–Wallis test.

**FIGURE 1:**
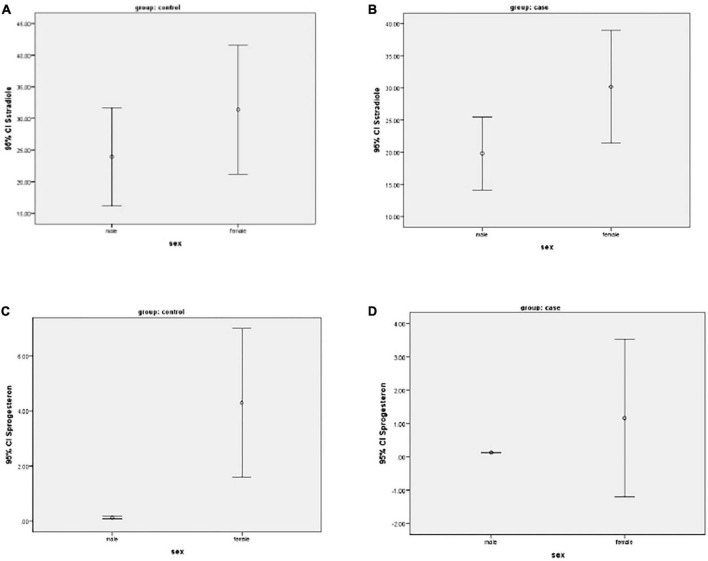
**(A–D)** Mean of progesterone and estradiol serum levels in two groups of men and women participants.

Figure 1 show progesterone and estradiol serum levels in men and women.

After including age and sex in the linear regression model, only the age for progesterone in the control group was statistically significant (Table 6).

**TABLE 6:** Linear regression coefficients on progesterone and estradiol serum levels to the age and sex of participants.

			Unstandardized coefficients		Standardized coefficients		
			**B**	**Standard error**	**Beta**	***t***	***P*-value**
Case group	Progesterone^1^	Age	-0.001	0.026	-0.008	−0.044	0.965
		Sex	1.043	0.561	0.324	1.86	0.073
	Estradiol^2^	Age	0.082	0.248	0.057	0.329	0.744
		Sex	10.125	5.446	0.323	1.859	0.073
Control group	Progesterone^3^	Age	-0.195	0.066	-0.497	−2.981	0.006
		Sex	1.528	2.113	0.121	0.723	0.475
	Estradiol^4^	Age	0.089	0.29	0.061	0.308	0.76
		Sex	8.665	9.337	0.184	0.928	0.361

^1^Adjusted *r*^2^: 0.04. ^2^Adjusted *r*^2^: 0.05. ^3^Adjusted *r*^2^: 0.26. ^4^Adjusted *r*^2^: −0.03.

## Discussion

Schizophrenia is a debilitating psychiatric disease. Many recent studies have reported differences in the severity and duration of the disease between the two genders, which have been attributed to the effect of sex hormones (24). Therefore, the present study aimed to compare estradiol and progesterone levels between patients with schizophrenia and their healthy counterparts.

Our results showed that the serum level of progesterone was significantly lower in schizophrenic patients than in healthy individuals, which was in agreement with some previous studies (16, 24, 25). These studies have noted a negative role for progesterone in developing the disease symptoms, predominantly negative symptoms, along with a link between estradiol and progesterone levels. Other studies have denoted a significant decrease in estradiol and progesterone levels in schizophrenic patients compared to control subjects, while other hormones have been noted to be elevated in these patients. Elevated levels of progesterone and estrogen in the luteal phase have been reported to correlate with milder symptoms, and a lower level of progesterone at the beginning of the treatment was noted to predict a better therapeutic response. The results of these studies are consistent with ours. Recent studies have highlighted the neuroprotective effects of progesterone, mediated *via* inducing anti-apoptotic mechanisms and promoting cell survival. Furthermore, this hormone has an inhibitory effect on glutamatergic synapses. Allopregnanolone, a progesterone metabolite, was shown to inhibit the dopamine-induced release of glutamate in the prefrontal cortex (3, 26).

Regarding the serum level of estradiol, our results revealed no significant difference between schizophrenic patients and control subjects. Contrary to our results, Bergemann et al. (27) showed that women with schizophrenia, with or without antipsychotic-induced hyperprolactinemia, had low serum estrogen levels. Also, Sánchez et al. denoted the stimulating effect of estrogen on the activity of dopaminergic neurons, especially in the striatum and nucleus accumbens (28, 29). Treatment with estradiol can regulate presynaptic and postsynaptic transmitters and receptors, as well as the synthesis, release, and circulation of dopamine in both cortical and striatal areas (30, 31). According to the studies mentioned, the lack of a significant difference in estrogen levels between the two study groups in the present study may be related to the confounding effects of antipsychotics, which could affect the dopaminergic pathway. Also, some studies have raised the role of racial differences in the variabilities observed in 17-beta estradiol and estrone levels, which may be related to various aromatase enzyme (32).

In the present study, no statistically significant positive correlation was observed between the average serum levels of estradiol and progesterone and the total scores of PANSS, Positive Positive and Negative Syndrome Scale (PPANSS), Negative Positive and Negative Syndrome Scale (NPANSS), and General Positive and Negative Syndrome Scale (GPANSS). Consistently, some studies have stated that the serum level of progesterone has a negative correlation with the total PANSS score and positive PANSS scores, suggesting a relationship between low hormone levels and the severity of the disease in schizophrenic patients (3).

In contrast with our study, in Weiser et al. study (2019) (33), which enrolled 200 female schizophrenic patients for eight weeks, one group received estradiol patches, and the other group was administered a placebo. The results showed noteworthy improvements in the positive symptoms of the disease according to the PANSS questionnaire, while younger participants did not benefit from estradiol. Likewise, Bergemann et al. assessed the relationship between estradiol level and the severity of positive and negative schizophrenia symptoms based on the PANSS and reported similar findings with a significant improvement in symptoms during the luteal phase (8, 34, 35). Although no significant difference was observed in the serum level of estradiol between the study groups in our study, it seems that estrogen exerts an antipsychotic effect, similar to other antipsychotics, on the symptoms of schizophrenia and, therefore, has a positive impact on the disease course in schizophrenic women. Contrary to our study, there have been studies that showed a negative correlation between serum progesterone level and PANSS total score and positive PANSS score, which indicates the relationship between low hormone levels and disease severity in schizophrenia patients.

## Conclusion

Considering the hormonal changes in schizophrenic patients compared to control subjects and their significant relationship with the severity of positive symptoms, determining hormonal levels in these patients and implementing complementary hormonal therapies such as estradiol and similar compounds can be beneficial as the starting point for schizophrenia treatment, where therapeutic responses can draw the future developmental framework.

### Limitations

The study limitations included the small sample size and the restrictions caused by the COVID-19 pandemic, demanding compliance with health protocols and, therefore, a reduction in visits to health centers, including psychiatric clinics, which prolonged the patient recruitment process and obtaining samples. Also, considering the effects of some drugs, such as risperidone and aripiprazole, on the level of the hormones assessed, it is necessary to adjust for the effects of such drugs in future studies. In addition, not considering sex differences intra-individual and ethnic biological variations were other limitation in the present study. A clinical trial study is suggested to investigate the difference hormone levels on schizophrenic patients.

## Data availability statement

The original contributions presented in this study are included in the article/supplementary material, further inquiries can be directed to the corresponding authors.

## Ethics statement

The studies involving human participants were reviewed and approved by the Ethics Committee of Golestan University of Medical Sciences (IR.GOUMS.REC.1400.346). The patients/participants provided their written informed consent to participate in this study.

## Author contributions

NS and ZS: conceptualization and methodology. MZ and DK: data curation, software, and writing – original draft preparation. AR: visualization and investigation. MZ and NS: supervision. DK and AR: validation. NS: resources. All authors reviewed and edited the manuscript and approved the submitted version.
